# Design, Synthesis, and In Vitro Evaluation of Hydroxybenzimidazole-Donepezil Analogues as Multitarget-Directed Ligands for the Treatment of Alzheimer’s Disease

**DOI:** 10.3390/molecules25040985

**Published:** 2020-02-22

**Authors:** Sílvia Chaves, Simonetta Resta, Federica Rinaldo, Marina Costa, Romane Josselin, Karolina Gwizdala, Luca Piemontese, Vito Capriati, A. Raquel Pereira-Santos, Sandra M. Cardoso, M. Amélia Santos

**Affiliations:** 1 Centro de Química Estrutural and Departamento de Engenharia Química, Instituto Superior Técnico, Universidade de Lisboa, Av. Rovisco Pais, 1049-001 Lisboa, Portugal; silvia.chaves@tecnico.ulisboa.pt (S.C.); s.resta7@studenti.uniba.it (S.R.); f.rinaldo@studenti.uniba.it (F.R.); marinamcosta91@gmail.com (M.C.); josselinromane@gmail.com (R.J.); karo.gwizdala@gmail.com (K.G.); 2 Dipartimento di Farmacia–Scienze del Farmaco, Università degli Studi di Bari “Aldo Moro”, Via E. Orabona 4, I-70125 Bari, Italy; luca.piemontese@uniba.it (L.P.); vito.capriati@uniba.it (V.C.); 3 Consorzio C.I.N.M.P.I.S., Via E. Orabona 4, I-70125 Bari, Italy; 4 CNC—Center for Neuroscience and Cell Biology, University of Coimbra, 3004-504 Coimbra, Portugal; araqpsantos@gmail.com (A.R.P.-S.); cardoso.sandra.m@gmail.com (S.M.C.); 5 Institute of Molecular and Cell Biology, Faculty of Medicine, University of Coimbra, 3000-548 Coimbra, Portugal

**Keywords:** hydroxyphenyl-benzimidazole, donepezil, anti-neurodegeneratives, Alzheimer´s disease, multifunctional drugs, metal chelation

## Abstract

A series of multi-target-directed ligands (MTDLs), obtained by attachment of a hydroxyphenylbenzimidazole (BIM) unit to donepezil (DNP) active mimetic moiety (benzyl-piperidine/-piperazine) was designed, synthesized, and evaluated as potential anti-Alzheimer’s disease (AD) drugs in terms of biological activity (inhibition of acetylcholinesterase (AChE) and β–amyloid (Aβ) aggregation), metal chelation, and neuroprotection capacity. Among the DNP-BIM hybrids studied herein, the structural isomerization did not significantly improve the biological properties, while some substitutions, namely fluorine atom in each moiety or the methoxy group in the benzyl ring, evidenced higher cholinergic AChE activity. All the compounds are able to chelate Cu and Zn metal ions through their bidentate BIM moieties, but compound **5**, containing a three-dentate chelating unit, is the strongest Cu(II) chelator. Concerning the viability on neuroblastoma cells, compounds **9** and **10** displayed the highest reduction of Aβ-induced cell toxicity. In silico calculations of some pharmacokinetic descriptors indicate that all the compounds but the nitro derivatives have good potential oral-bioavailability. Overall, it can be concluded that most of the studied DNP-BIM conjugates showed quite good anti-AD properties, therefore deserving to be considered in further studies with the aim of understanding and treating AD.

## 1. Introduction

Alzheimer’s disease (AD) is a severe age-dependent neurodegenerative disorder, with no cure so far, characterized by a drastic progressive decline in memory and cognitive abilities. With the increase of life expectancy of humans, the number of AD patients is growing dramatically and in 2050 it will be around 150 million worldwide [1]. In spite of all the research performed to understand and treat AD, there has been a high rate of failure in AD drug development programs [2,3]. The currently available therapies consist of acetylcholinesterase (AChE) inhibitors, such as donepezil (DNP, Aricept^®^), and an *N*-methyl-d-aspartate receptor (NMDAR) antagonist (memantine) [4]. However, they can only lead to some temporary enhancement of neurotransmission levels (e.g., cholinergic and glutamatergic), and so a valid therapy for AD remains an urgent unmet medical need. From the research efforts to understand this disease, several factors have been considered to play important roles in the pathophysiology of this illness, such as deficits of acetylcholine, β-amyloid deposits, τ-protein phosphorylation, oxidative stress, and metal dyshomeostasis [5]. The disease complexity has been recognized as the main cause for clinical failures of drugs based on the individual pathogenic targets. Therefore, over the last decade, the need to design chemical tools capable of interacting with multiple pathological elements of AD has prompted the scientific community and led to extensive research on multi-target-directed-ligands (MTDLs) [6,7,8]. Under this strategy, many multifunctional molecules have been discovered with the aim to combine the control of the symptoms with real disease modifying actions. Thus, various approved acetylcholinesterase drugs have been repositioned for further derivatizations, by enclosing or fusing other molecular entities to interact with some other AD targets, namely those related with the toxic amyloid plaque, such as the inhibition of β-amyloid (Aβ) fibril formation (e.g., inhibition β-Secretase, BACE-1) and its aggregation, as well as the control of effects of oxidative stress and metal dyshomeostasis, due to their recognized role in deleterious protein/peptide modification and aggregation [9,10,11,12]. In particular, several DNP derivatives have been recently developed to associate the cholinesterase inhibition with anti-oxidant capacity or with anti-β amyloid cascade properties [13,14]. Also, the implication of metal ions, such as copper, zinc, and iron, has long been recognized in AD, associated to the enhancement of deleterious reactive oxygen species (ROS) in the brain (Fe, Cu) and to intertwined roles (Cu, Zn) in β-amyloid aggregation [15,16,17]. Therefore, intensive research has recently been dedicated towards the development of MTDLs enclosing metal chelating functions, as potential anti-AD drug candidates (for reviews see refs [5,10,18,19]). 

**Figure 1:**
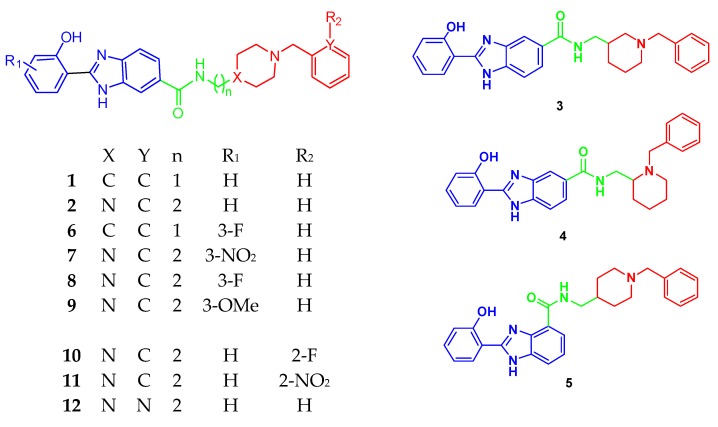
Structures of the studied donepezil-hydroxyphenylbenzimidazole (DNP-BIM) hybrids.

This subject has also been one of the main recent aims of our research group, with MTDLs enclosing a molecular fragment based on AChEi drug (e.g., Tacrine, DNP), which has been derivatized with a variety of functional moieties, several of them including metal chelating groups (such as cysteine, hydroxypyridinone, hydroxycinamate, hydroxybenzofurane, and hydroxyphenylbenzimidazole (BIM)) [19,20,21,22]. The interesting properties revealed by the recently reported novel tacrine-hydroxyphenylbenzimidazole (TAC-BIM) hybrids [22] led to the development of other MTDL analogues, by attachment of a BIM group to DNP active mimetic ligands (benzyl-piperidine/benzyl-piperazine), to assure AChEi, inhibition of Aβ aggregation and modulation of metal dyshomeostasis [23]. As a continuation of our efforts to search for new DNP-BIM hybrids, herein we present a series of analogues (see Figure 1), which aims to explore the effect of positional isomerization and introduction of a substituent on each of the above referred main multiple anti-AD properties as well as on drug-likeness prediction. Discussion is also provided by establishing comparison with previously reported DNP-BIM hybrids. 

## 2. Results and Discussion

### 2.1. Molecular Design and Docking 

Taking into account the recognized medical interest on the donepezil (DNP) drug to ameliorate AD symptoms, it was our inspiration to select *N*-benzylpiperidine and its isosteres benzylpiperazine and benzylpyridine as the main pharmacophores in the design and development of new hybrid compounds with multitarget capacity. The second pharmacophore herein explored is hydroxyphenylbenzimidazole (BIM), aimed to mimic the indanone scaffold of DNP, and also to provide metal-chelating and anti-Aβ aggregation capacity, similarly to other hybrid analogues recently reported [22,23]. Thus, the *N*-benzylpiperidine and its isostere moieties should account to the most important binding interaction of DNP within the catalytic anionic site (CAS) of AChE [10], while the BIM moiety should mimic the indanone moiety in DNP and reinforce the interaction with AChE through its extra-binding to the peripheral active site (PAS), thus enabling a dual binding mode. 

**Figure 2:**
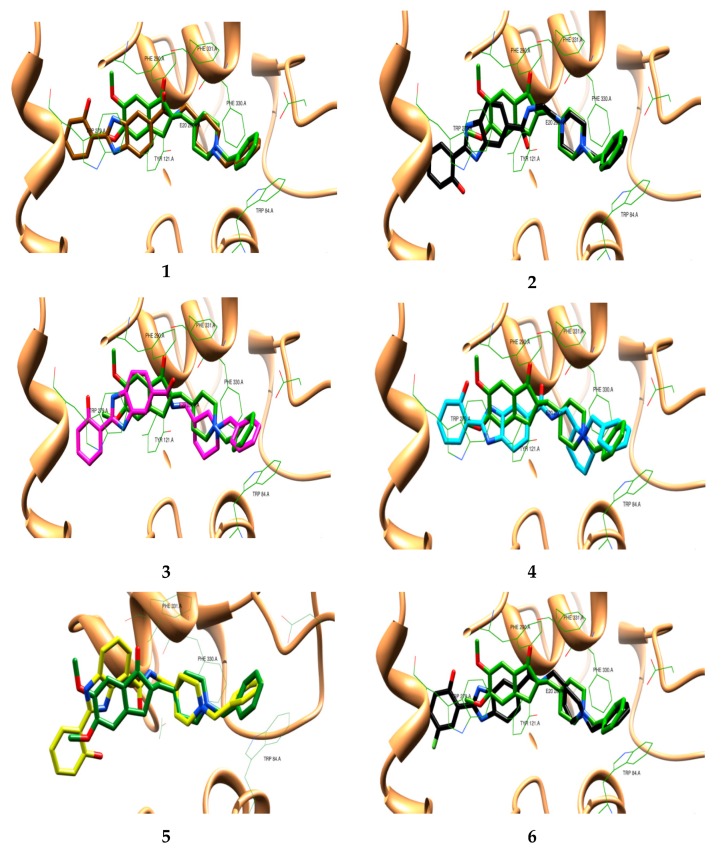

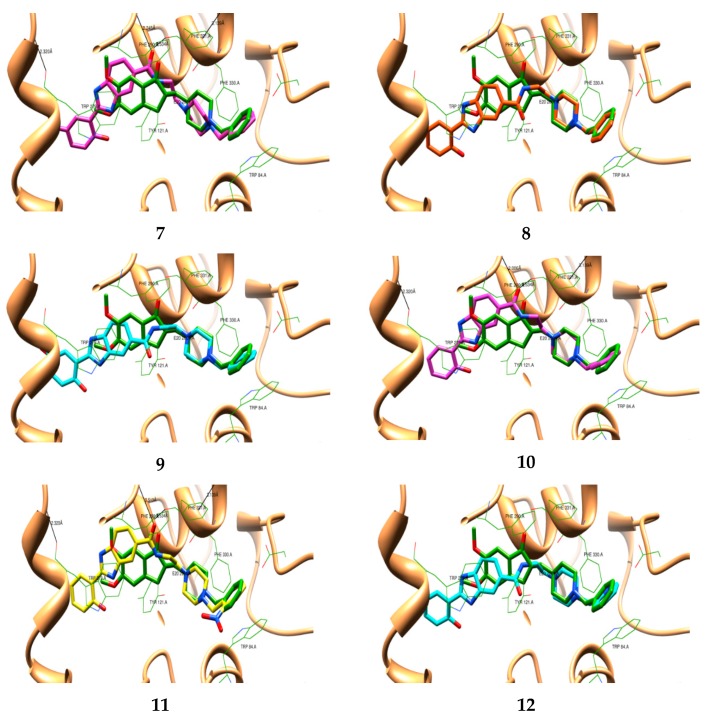
Superimposition of the different DNP-BIM hybrids and the original ligand (donepezil-green), as a result of their docking inside the acetylcholinesterase (AChE) active site (PDB code 1EVE) [24].

Docking studies were performed with this set of new hybrids in order to gain some insight into the differences in their binding interactions with the active site of AChE. Specifically, it was intended to understand the effect of positional isomerization and of specific substitutions in the benzylpiperidine, benzylpiperazine, and hydroxyphenylbenzimidazole (BIM) moieties on the ligand-enzyme binding, namely in the dual binding mode. Similarly, as previously reported for compounds **1** and **2**, the docking simulations of this set of hybrids were performed with AChE (PDB code: 1EVE) [24], using the X-ray crystal structure of the *Tc*AChE complex with DNP. Firstly, the co-crystallized ligand (DNP) was docked back into the binding site of the enzyme and superimposed with the native ligand (Figure S1). Being satisfied with the good overlay, the same docking protocol was used for further docking studies with the target compounds. The best docked poses of each synthesized compound were selected and superimposed with that of DNP, as shown in Figure 2. Similar to the already published simple analogues (**1**, **2**), they showed a pattern of orientation resembling that of DNP. In particular, all the compounds have the first pharmacophore moieties, namely the benzyl-piperidinium (**3**–**6**), -piperazinium (**7**–**11**), or -pyridinium (**12**), anchored to the CAS of AChE (bottom of the gorge), through binding interactions via aromatic π-π stacking with the phenyl ring from Trp 84A; at the middle of the gorge, the charged nitrogen atom can establish a cation–π binding interaction with the phenyl group of Phe330. Noteworthy is the observation that the benzyl-piperidinium moieties of positional isomers (**3–5**) present some bending relative to that of DNP, though no significant effect appeared on the complexes with the benzyl-piperazinium substituted (**10**, **11**) or the benzyl-pyridinium (**12**) moieties. Regarding the BIM moieties, similar to the DNP indanone ring, these moieties are at the entrance of the gorge interacting via π-π stacking with the peripheral anionic residue Trp279. However, these docking simulations indicate for the BIM moieties less favorable interactions with PAS than DNP, which can be mainly attributed to the fact that the tricyclic BIM moieties are longer than the bicyclic indanone moiety of DNP. Furthermore, some additional distortion seems to occur for positional isomers (**3–5**) and substitution with big size substituents (-NO_2_, **7**; -OMe, **9**). In fact, the AChE inhibition assays herein performed (see Section 2.4.1) confirm lower inhibitory activity for **4**, **5**, **7**, and **11**, probably due to the existence of distorted configurations of these compounds inside the active site of the enzyme. Interestingly, the substitution by the small size fluorine atom (**8**, **10**) does not seem to contribute to any further distortion, as compared with the simple analogues (**1**, **2**), though from the docking studies we cannot observe any engaged positive binding interactions. 

### 2.2. Synthesis

**Scheme 1:**
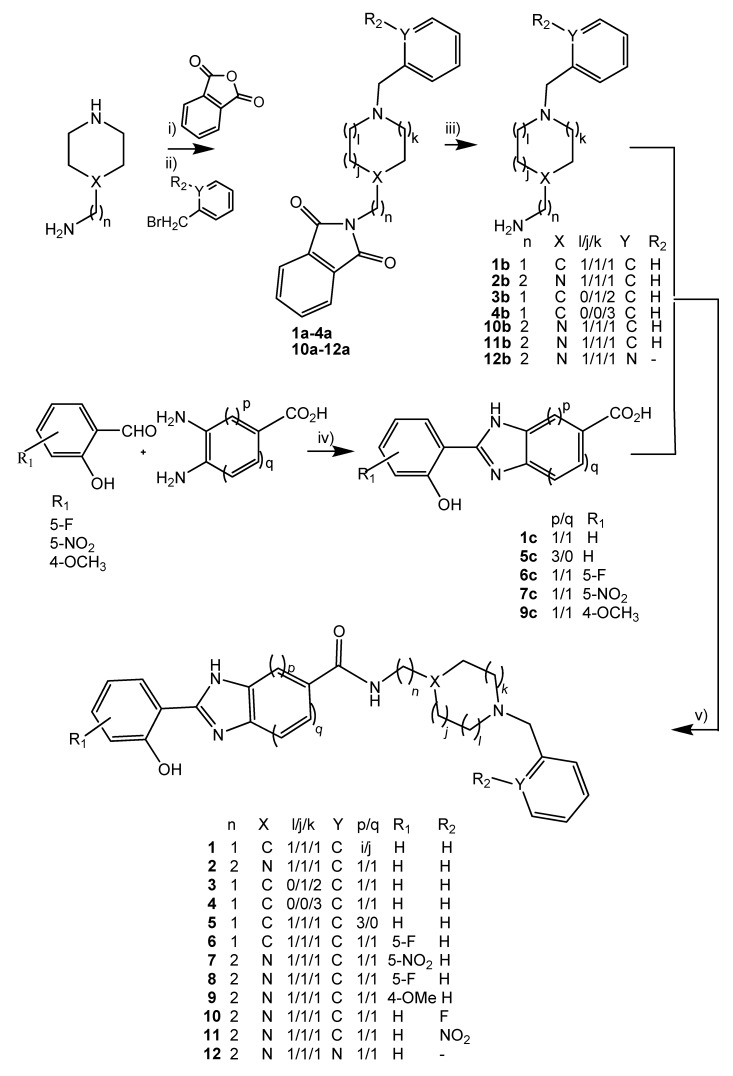
Reagents and conditions: (i) Phthalic anhydride, net, 180 °C, 4 h; (ii), TEA, K_2_CO_3_, ACN, 50 °C, 3 h; (iii) N_2_H_4_.xH_2_O; (iv) (A) Na_2_S_2_O_5_, DMA, 100 °C, 12 h; (B) Na_2_S_2_O_5_, ChCl/Gly, 50 °C, 30 min; (v) DCC, *N*-hydroxysuccinimide, dry DMF, RT, 40 h.

Twelve hybrid compounds resulted from the conjugation of a hydroxyphenylbenzimidazole (BIM) moiety with benzylpiperidine (PP, **1**, **3**−**6**), benzylpiperazine (PZ, **2**, **7**−**11**), and also benzylpyridine (**12**). The hybrids were obtained by following the convergent synthetic approach depicted in Scheme 1. The preparation of each final intermediate moiety, **b**-series of benzyl amine derivatives (**1**–**4b** and **10**–**12b**) and ***c***-series of carboxyl BIM derivatives (**1c**, **5**–**9c**), involved mostly procedures previously reported [22,23]. More specifically, the synthesis of the benzyl-cyclic amino derivatives (***b***-series) involved a first one-pot-two-step reaction, with preliminary protection of the primary alkylamine group to a phthalimide group by its reaction with phthalic anhydride (FA) in absence of solvent and subsequent benzylation of the secondary cyclic amine with benzyl bromide in dry acetonitrile under basic conditions; the final deprotection of the primary amine was performed with hydrazine hydrate in refluxing ethanol. The preparation of the ***c***-series of intermediates, hydroxyphenylbenzimidazole acid derivatives, involved the cyclization of diaminobenzoic acid derivatives with diverse salicylaldehydes, in the presence of sodium metabissulfite and *N*,*N*-dimethylacetamide (DMA) (method A) [22]. However, for some compounds (**6c**, **7c**, **9c**), an eutectic solvent mixture, choline chloride/glycerol (ChCl/Gly), was used (method B) instead of DMA, with improved effectiveness [25]. The last reaction step involved the coupling of amine intermediate moieties (***b***-series) with carboxylic intermediate moieties (***c***-series), via amide formation, and it was carried out in dry DMF, using NHS and DCC as activators.

### 2.3. Metal Chelation 

In order to evaluate the chelating capacity of this type of compound towards Cu(II) and Zn(II), some of them (**7** and **12**) were selected and their acid-base behavior was firstly studied. A working medium of 50% (*w*/*w*) DMSO/water was chosen due to solubility reasons and also to enable the comparison with results for other hybrids, previously reported in the same medium. Although a 50% DMSO/water (*w*/*w*) medium has been implemented to perform these solution equilibrium assays, in terms of cellular studies the concentration of ligand employed is significantly lower (<7 μM) and therefore the final concentration of DMSO used in culture media would be inferior (<1%) and with no associated modifications in biological tissues. 

**Figure 3:**
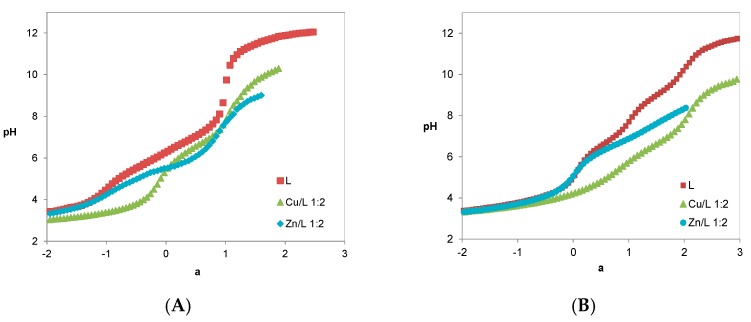
Potentiometric titration curves of compounds **7** (**A**) and **12** (**B**) (*C*_L_ = 6.7 × 10^−4^ M, a represents moles of added base per mole of ligand). Experimental conditions: 50% *w*/*w* DMSO/water, *I* = 0.1 M KCl, *T* = 25.0 ± 0.1 °C.

The protonation constants of the compounds were determined by potentiometry (see Figure 3 and Table 1) by using Hyperquad [26]. 

Compound **7** was isolated in its neutral mono-protonated form (HL, a = 0 in Figure 3), although it has three dissociable protons (H_3_L^2+^), while compound **12**, with four dissociable protons (H_4_L^3+^), was isolated in its mono-positive charged di-protonated form (H_2_L^+^, a = 0 in Figure 3). Table 1 shows three values of protonation constants (log *K*_i_ = 6.99, 5.51, and 2.95) calculated for compound **7** and four values for compound **12** (log *K*_i_ = 9.09, 6.44, 3.64, and 3.17). 

^1^H NMR titration studies previously reported revealed that, for compounds **1** and **2**, the sequence of protonation corresponds to the following order: firstly, the phenolic oxygen of the BIM moiety, afterwards the *N*-piperazine nitrogen (opposite to benzyl group)/*N*-piperidine nitrogen atom, and lastly the imidazole nitrogen *N*(3) of the BIM moiety. Concerning compound **7**, with analogous sequence of protonation, both log *K*_1_ (6.99) and log *K*_2_ (5.51) are much lower than the corresponding ones for compound **2** (see Table 1). The decrease of log *K* value corresponding to the phenolic oxygen is attributed to the resonance electron withdrawing nature of the nitro group present at the *para* position (e.g., phenol 9.98 [27] and 4-nitrophenol 7.15 [28]), that stabilizes the conjugated base. Regarding compound **12**, the first two and the fourth protonation constants correspond to those of the analogous compound **2**, while the third one (log *K*_3_ = 3.64) is attributed to the pyridine nitrogen; the decrease of the log *K* value, when compared with that of pyridine (5.24, in water [29]), may be due to the electron withdrawing nature of the neighbor nitrogen atom from the piperazine moiety, that stabilizes the conjugated base by resonance effect. Finally, for all the hybrids contained in Table 1, the decreasing of the protonation constants corresponding to the N(3) atom (log *K*_3_ = 2.86−3.37) and *O*-phenol (log *K*_1_ = 6.99−9.09), when compared with equivalent standard groups (e.g., imidazole 6.95 [30] and phenol 9.98 [27]), reflects the existence of intramolecular hydrogen bond interactions involving these atoms in the BIM moiety. 

Regarding compound **5** (see Table 1), previously studied [31], its three protonation constants and sequence of protonation were found to be similar to those of compound **1**.

The metal chelating ability of hybrids **7** and **12**, due to the BIM bidentate unity with (*N*,*O*) coordination mode, was studied by potentiometry. Figure 3A,B show a change in the deprotonation profile of the ligand titration curves due to the presence of the metal ions. In fact, the curves for the Cu(II)/L systems lie well below that of the ligand, while for the Zn(II)/L systems stay under for a > −1 (compound **7**) or a > 0 (compound **12**), thus supporting the formation of 1:1 and 1:2 M/L ratio complexes with the deprotonated and mono-protonated forms of the ligand and with relative metal dependent stability order (Cu > Zn). Table 1 contains the equilibrium models obtained from the fitting analysis of the potentiometric curves by program Hyperquad [26]. In these equilibrium models, which the coordination core of the compounds integrates the bidentate (*N*,*O*) BIM moiety, MHL and MH_2_L_2_ correspond to species with the ligand containing one protonated nitrogen atom at the piperazine moiety, ML and ML_2_ correspond to complexes involving the completely deprotonated form of the ligand while MH_-1_L_2_ are mixed ligand-hydroxo metal complexes. 

**Figure 4:**
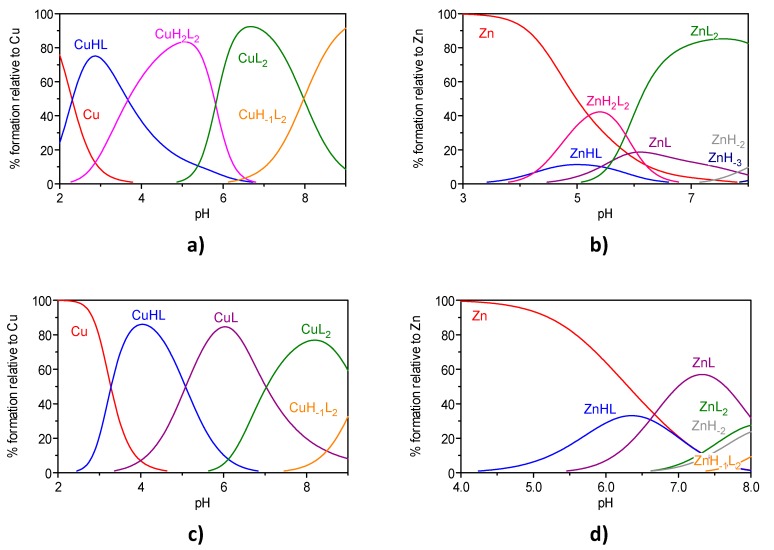
Species distribution curves for the 1:2 M(II)/L systems with compound **7** and **a**) M = Cu, **b**) M = Zn, as well as with compound **12**, and **c**) M = Cu, **d**) M = Zn (*C***_L_** = 6.7 × 10^−4^ M).

Figure 4 shows the species distribution curves for the 1:2 M(II)/**L** (M = Cu, Zn) systems, involving compounds **7** and **12**, by using the potentiometric determined model at the used experimental conditions. 

The species distribution curves show that, for the concentrations used in the experimental conditions, the metal complexation with compounds **7** and **12** starts at pH around 2 for Cu(II) or above 3.5–4 for Zn(II), with the formation of MHL species. Moreover, both 1:1 (MHL, ML) and 1:2 (MH_2_L_2_, ML_2_, MH_-1_L_2_) species are formed and the mixed hydroxo-ligand complex (MH_-1_L_2_) starts to appear above pH 6–7. For the Zn(II)/L systems, some competition with metal hydroxide species occurs above pH 6.5–7. Previous results from UV-VIS spectral data collected for the analogous compounds **1** and **2** pointed towards a chelating core composed by the *N*(3) imidazole and *O*-phenol atoms of the ligands, excluding the hypothesis of metal coordination through the distant nitrogen atoms of piperidine or piperazine [33]. Therefore, it is expectable that the same coordination core is kept for hybrids **7** and **12**. 

Comparison of the metal chelating capacity of compounds **7** and **12** with that of the already studied analogous DNP-BIM hybrids (see Table 1) reveals that these kind of ligands are good binders of Cu(II) and only moderate for Zn(II). In fact, the calculated pM values (at pH 7.4, *C*_L_/*C*_M_ = 10, *C*_M_ = 10^−^^6^ M) for the set of DNP-BIM hybrids studied are higher for Cu(II) (pCu = 10.4−14.3) than for zinc (pZn = 6.3−6.5). Moreover, by comparing the pM values relative to **7** and **2**, it is possible to see some influence of the *p*-nitro group on the increase of stability of the metal complexes, although compound **7** has lower total basicity than **2**. Noteworthy is also the higher chelating capacity towards Cu(II) of compound **5** vs. **1** (pCu (**5**) = 14.3, pCu (**1**) = 10.7), attributable to the possibility of compound **5** being able to establish a tridentate (*N*,*O*,*O*) coordination to the metal ion, by including the adjacent carbonyl oxygen atom [30], with correspondingly higher stability of its copper complex. 

### 2.4. Biological Studies in Solution

#### 2.4.1. Acetylcholinesterase (AChE) Inhibition

The inhibition of AChE by the DNP-BIM hybrids herein studied was assayed by using a variation of Ellman´s test [23,34] and the IC_50_ values obtained are included in Table 2. All the inhibitors, but the benzyl-pyridinium derivative (**12**), present IC_50_ values in low micromolar range. Among the piperidine (PP) series (PP-BIM), the best results were obtained with compounds **1**, **3**, and **6**, while for the piperazine (PZ) series (PZ-BIM), the most potent were compounds **8**, **9**, and **10**. Although the present hybrids are weaker AChE inhibitors than DNP (IC_50_ = 0.026 μM), this does not weaken the proposed drug design since the advantages of using a multi-target drug in multifactorial diseases can surpass the drawback of losing some specific property of a single-target drug.

**Figure 5:**
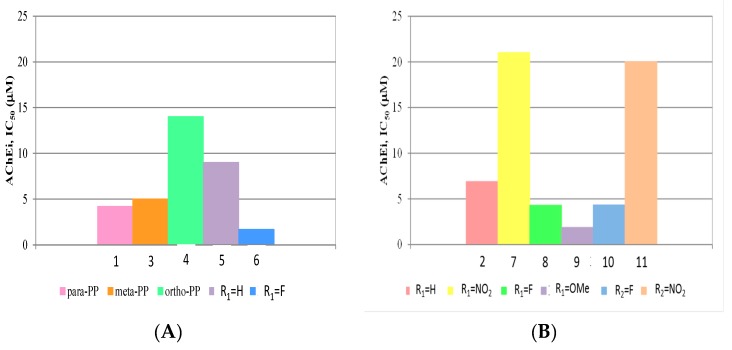
Graphical summary of the effect of different structural parameters on the inhibition of AChE (AChEi, IC_50_) for: (**A**) PP-BIM hybrids; (**B**) PZ-BIM hybrids.

In order to understand the effect of different structural changes on the inhibitory capacity of AChE such as the existence of positional isomers (compounds **1**, **3**, and **4**) or the inclusion of R_1_ or R_2_ substituents, a brief structure-activity analysis can be performed based on graphical representation of the enzymatic activity (IC_50_) (Figure 5). 

Analysis of the data illustrated in Figure 5A (or Table 2), shows that the inhibitory capacity of the positional isomers on the PP moiety is according to the following sequential order: *para* (**1**) > *meta* (**3**) > *ortho* (**4**). Concerning the attachment point in the BIM moiety, the *para* position (**1**) is also preferred to the *ortho* one (**5**). Therefore, apart from compounds **3**, **4**, and **5**, this series of DNP-BIM hybrids is mainly composed by positional *para* isomers, both in the PP and in the BIM moieties, aimed to be better accommodated in the enzyme structure and so with higher inhibitory activity against AChE. Figure 5A also shows that the inclusion of a fluorine in the BIM moiety leads to an activity improvement e.g., **1** versus **6**). 

On the other hand, Figure 5B shows the effect of substituent groups, as R_1_ in the BIM moiety or R_2_ in the benzyl of the PZ unity. In both types of substitutions, it is evident that the fluorine (and also R_1_ = -OMe) leads to enhancement of the inhibitory capacity, while the nitro group decreases its value. 

Overall, the best AChEi activity was achieved for *para*-/*para*- hybrids **6** and **9**, with fluorine and methoxy R_1_ substituents in the BIM moiety, respectively, which demonstrates the determinant role of these substituents in the establishment of interactions within the active site of AChE.

#### 2.4.2. Inhibition of Aβ_1–42_ Aggregation

The anti-amyloidogenic capacity of the compounds was also evaluated in vitro, in the absence and presence of Cu(II), based on the measurement of the fluorescence emission of Thioflavin T (ThT), a stain known by its A*β* fibril binding capacity [36,37]. This binding interaction can be analyzed by fluorimetry, since the presence of ThT-fibrils increases the absorbance and the emission of the ThT dye, and it also induces red shifts on the absorbance (from 385 to 446 nm) and emission peaks (from 445 to 485 nm) [22]. All the measurements were performed after incubation (24 h, 37 °C) of the self-mediated and Cu^2+^-induced Aβ aggregates in the presence/absence of the compounds under evaluation.

In fact, it is well known that Aβ binds Cu(II) and, although this interaction has been associated to the induction of Aβ aggregation [15,16], it has also been admitted that it can lead to the precipitation of amorphous deposits of the peptide and not to ThT-positive β sheet rich amyloid fibril formation with different studies being performed on the analysis of the effect of Cu(II) on the propensity for Aβ fibril formation as well as on the effect of metal chelators on this process [38,39]. Several reported fluorescence studies based on the ThT dye have been performed in different experimental conditions (solvent used for Aβ, pH, and incubation time), which turn difficult comparison of results. Under our experimental method it was observed a tendency for decreasing the fluorescence intensity for Aβ in the presence of copper, in comparison with its absence, which may be due to some precipitation of amorphous deposits of the peptide rather than formation of β sheets [38,39]. In former studies, with TAC-BIM derivatives (**1**, **2**) [22,23,33], a fluorescence-independent method like transmission electron microscopy (TEM) was used, due to possible quenching interferences in the emission of the paramagnetic copper ion, and it was observed that scarcer aggregates appear in the presence of Cu(II) when comparing Aβ with Aβ + Cu(II). Furthermore, it has been proposed that metal chelators might either dissolve the deposits in the brain tissue or prevent Aβ aggregation. 

**Figure 6:**
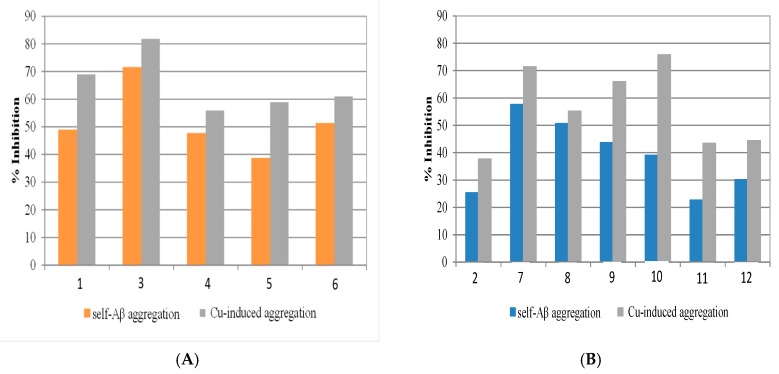
Graphical summary of the effect of structural parameters on the inhibition of Aβ aggregation (self- and Cu-induced, in %) for: (**A**) PP-BIM hybrids; (**B**) PZ-BIM hybrids.

Herein, due to solubility problems of some compounds in the phosphate buffer (0.215 M, pH 8.0) of the working medium, the assays were carried out in the presence of 40 μM concentration of inhibitor and 20 μM in the case of compound **4**, instead of 80 μM as previously reported [33]. The obtained data, expressed as percentage of aggregation inhibition, is included in Table 2 and graphically presented in Figure 6. The results show that all the compounds are able to inhibit Aβ aggregation with good-to-moderate activity. The activity dependence on ligand structural features may be mainly explained by the different ability for intercalation into the fibrils, as already assumed for other inhibitors [22,40]. Among the hybrids under study, **3** exhibited the highest inhibitory activity for self- and Cu-induced Aβ aggregation, although very good inhibitory activity is also presented by **1**, **6**, and **7**. The data obtained for compounds **6** and **7** indicate that the presence of R_1_ = F, -NO_2_ substituents in the BIM have some small positive effect on the inhibition of Aβ aggregation. On the other hand, compounds **5**, **10**, **11**, and **12** showed decreased inhibitory activity, which may result from a somehow more difficult intercalation into the fibrils, when there is an *ortho*-attached BIM or when the PZ benzyl unity is substituted.

Concerning the inhibition of Cu-induced Aβ aggregation, all the compounds induce an increase when comparing with their corresponding capacity for self-inhibition, in accordance with their good chelating capacity towards copper. Nevertheless, these chelators have conditional dissociation constants (*K*´_d_) for the copper complexes at pH 7.4 of 34 nanomolar (**1**), 24 nanomolar (**2**), 254 picomolar (**5**), 80 nanomolar (**7**), and 365 picomolar (**12**). These values are outside the proposed range *K*´_d_ = 1−10 picomolar corresponding to chelators eventually able to retrieve copper from A*β* peptide (*K*´_d_ = 10 picomolar–100 nanomolar for Cu(Aβ) complexes) [41]. Therefore, it seems that there is no competition for copper between these BIM hybrids and Aβ peptide, and so the inhibition process must be mainly due to ligand intercalation between the β-sheets of Aβ fibrils. TEM assays previously performed by us on analogous BIM hybrids [22,23,33] also confirmed the role of these kinds of compounds in the reduction of Aβ aggregation, both in the absence and presence of copper. 

### 2.5. Cell Viability and Neuroprotection

**Figure 7:**
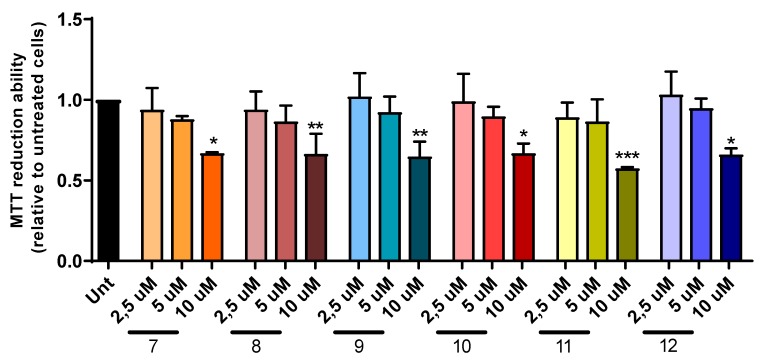
Dose-response screening to select non-toxic concentrations of compounds **7**–**12**. Cells were treated with different concentrations of the compounds for 24 h and MTT reduction assay was performed to evaluate cell viability. Results are expressed relatively to SH-SY5Y untreated cells, with the mean ± standard error of the mean (SEM) derived from four different experiments. * *p* < 0.05; ** *p* < 0.01; *** *p* < 0.001, significantly different when compared with SH-SY5Y untreated cells.

The neuroprotective effect of DNP-BIM hybrids designed for AD was evaluated using SH-SY5Y cell line treated with Aβ_1–42_ or ferrous sulfate and *L*-Ascorbic acid (Fe/Asc). A dose-response curve was performed to select a non-toxic concentration of each compound (Figure 7). For all the compounds, a concentration of 2.5 µM showed to be the highest non-toxic value.

**Figure 8:**
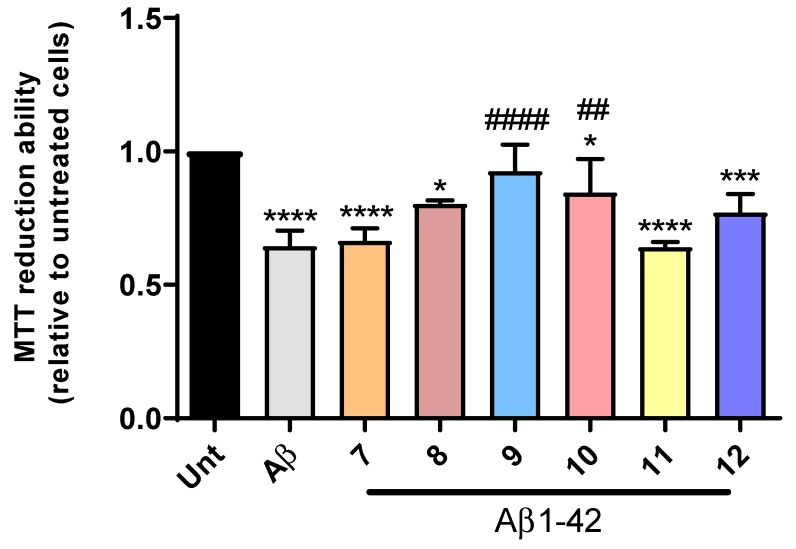
Neuroprotective effect of compounds **7**–**12** from Aβ_42_-induced toxicity on SH-SY5Y cells. Cells were treated with Aβ_42_ peptide (1 µM) for 24 h in the presence or absence of the compounds (1 h pre-incubation plus 24 h co-incubation). Evaluation of cell viability was performed using the MTT reduction assay. Results are expressed relatively to SH-SY5Y untreated cells, with the mean ± SEM derived from four different experiments. * *p* < 0.05; *** *p* < 0.001; **** *p* < 0.0001, significantly different when compared with SH-SY5Y untreated cells; ## *p* < 0.01; #### *p* < 0.0001, significantly different when compared with Aβ_42_ treated SH-SY5Y cells. The concentration used for all the compounds was 2.5 µM.

**Figure 9:**
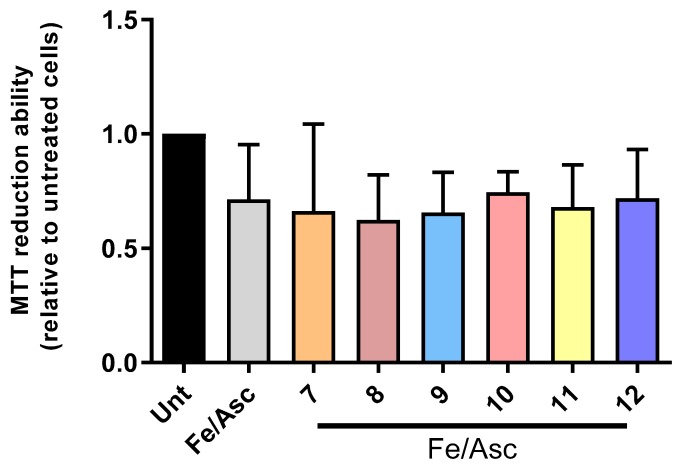
Neuroprotective effect of compounds **7**–**12** from *L*-Ascorbic Acid (AscH (-))/Ferrous sulphate (Fe) toxicity on SH-SY5Y cells. Cells were treated with Fe/Asc (2.5 mM and 5 mM, respectively) for 24 h in the absence or in the presence of the compounds (1 h pre-incubation plus 24 h co-incubation). Evaluation of cell viability was performed using the MTT reduction assay. Results are expressed relative to SH-SY5Y untreated cells, with the mean ± SEM derived from four different experiments. The concentration used for all the compounds was 2.5 µM.

Extracellular formation of senile plaques of aggregated Aβ along with the production of reactive oxygen species (ROS) are processes involved in the neurodegeneration that characterize AD [42]. Herein, we observed that Aβ induces a decrease in cell viability close to 50% and, remarkably, compounds **9** and **10** prevented Aβ-induced cell toxicity (Figure 8). Additionally, ROS production is also one of the hallmarks associated with AD pathogenesis [42]. To study the neuroprotective effect of DNP-BIM hybrids against oxidative stress, we incubated our cell model with Fe/Asc, which showed a decrease of 25% in the cell viability when compared with untreated cells (Figure 9). However, none of the compounds presented a statistically significant neuroprotective effect by preventing ROS production, even the compounds **9** and **10** that showed to be able to inhibit Aβ aggregation.

### 2.6. Prediction of Pharmacokinetic Properties

To get some insight on the drug-likeness of the studied hybrid compounds, *in silico* calculations of some pharmacokinetic descriptors were performed by the QikProp program [43]. Analysis of the selection of calculated parameters depicted in Table 3 shows that almost all the compounds present no violations of Lipinski’s rule of five [44], which can be indicative of their potential oral-bioavailability. However, compounds **7** and **11**, with a nitro substituent, present a molecular weight (MW) slightly higher than 500 Da (516.55 and 500.55), resulting in one violation of that rule. The range of values (2.586−4.513) calculated for log *P* (*c*log *P*, octanol-water partition coefficient) are within the normal range of values calculated for drugs (−2 to 6.5) [43], although hydrophilic character is generally higher for the piperazinic derivatives (X = N), as compared with the piperidinic analogues (X = C). To assess the capacity of the compound to cross the blood-brain barrier (BBB), log BB was calculated. The range of values obtained for log BB are also within the range calculated for drugs (−3 to 1.2) [43], although it is generally accepted that compounds with log BB < −1 are poorly distributed in the brain and so with low CNS activity. Regarding the Caco-2 permeability, the values obtained for the piperidinic derivatives are higher than for the piperazinic analogues (<100), thus indicating for this last series of compounds a more moderate absorption from the intestinal tract to the blood. Overall, the remarkably low values obtained for Caco-2 permeability of the nitro-derivatives (<10), together with the lowest values for *c*log *P* and log BB, suggest that these hybrids should be excluded from further drug development. 

Overall, excepting the nitro-derivatives, generally, this set of hybrids presents well balanced lipophilic/hydrophilic character and moderate blood-brain barrier permeability (log BB), thus making them eligible as potential drug candidates.

## 3. Materials and Methods

### 3.1. Chemicals

For the synthesis, all the reagents (from Sigma-Aldrich, St. Louis, MO, USA, Alfa-Aesar, Kandel, Germany, Acros, Thermo Fisher Scientific, Geel, Belgium) were of analytical grade and used as supplied. Whenever necessary, the solvents were dried according to standard methods [45]. The chemical reactions were followed by thin layer chromatography (TLC) using alumina plates coated with silica gel 60 F254 (Macherey-Nagel, Düren, Germany). Column chromatography purifications were performed on silica gel (Merck 230–400 mesh (Geduran Si 60Darmstadt, Germany). The purity of the compounds was assessed by determination of the melting points (M.P.) and by using NMR and mass spectral techniques. The melting points were measured with a Leica Galen III (Microsystems, Wetzlar, Germany) hot stage apparatus and are uncorrected. ^1^H- and ^13^C-NMR spectra were recorded either on Bruker AVANCE III-300 (300 MHz and 75.5 MHz) or Bruker AVANCE III-400 (400 MHz and 100 MHz) NMR spectrometers (Billerica, MA, USA), at 25 °C. Chemical shifts are reported as *δ* values (ppm) from internal reference TMS (tetramethylsilane, Sigma-Aldrich, St. Louis, MO, USA) and coupling constants (*J*) in Hertz. The following abbreviations have been used: s = singlet, d = doublet, t = triplet, m = multiplet. The mass spectra were performed on a 500 MS LC Ion Trap mass spectrometer (Varian Inc., Palo Alto, CA, USA) equipped with an ESI ion source, operated in the positive ion mode. 

### 3.2. Synthesis of the Compounds

#### 3.2.1. Synthesis of the Benzyl-Piperidine as Well as Benzyl- and Pyridinyl-Piperazine Alkylamino Derivatives (**1**–**4b**,**10**–**12b**)

##### 3.2.1.1. Synthesis of the *N*-benzyl Compounds with Isoindoline-1,3-Dione Protection (**1**–**4a**, **10**–**12a**)

###### General Procedure

Phthalic acid anhydride (1 eq) and the aminoalkyl-piperidines or -piperazine derivatives (i-(aminomethyl)piperidine (i = 2,3,4) or 1-(2-aminoethyl)piperazine (1 eq) were heated at 160 °C for 4 h. The resulting dark brown solid (1 eq) was mixed with K_2_CO_3_ (6.6 eq), triethylamine (1.4 eq), and benzyl bromide derivatives or 2-(bromomethylpyridine (2.3 eq), and refluxed in acetonitrile for 24 h. Then, the mixture was cooled at rt, filtered and to the organic phase was added water, and then extracted with ethyl acetate (three times). The organic layers were collected, dried over anhydrous sodium sulphate, and concentrated under reduced pressure. The crude material was purified through chromatography column (with eluent: DCM/MeOH/TEA (98:2:0.1) or ACN/H_2_O (7:1)) or recrystallization. 

*2-((1-Benzylpiperidine-4-yl)methyl)isoindoline-1,3-dione* (**1a**). Starting from 4-(aminomethyl)piperidine and afterwards benzyl bromide the a pure title compound was obtained as a yellow solid (η = 40%), M.P. = 129−132 °C). ^1^H RMN (300 MHz, DMSO-*d*_6_), δ (ppm): 1.24−1.38, 1.52−1.91, 3.44−3.50 (m, 9H, piperidine), 2.73 (d, 2H, *J* = 12.0 Hz, N*CH_2_*CH), 3.41 (s, 2H, N*CH_2_*Ph), 7.15−7.40 (m, 5H, *Ph*), 7.79–7.91 (m, 4H, *PhTh*). M/z (ESI-MS): 335 (M + 1)^+^.

*2-(2-(4-Benzylpiperazin-1-yl)ethyl)isoindoline-1,3-dione* (**2a**)**.** Starting from 1-(2-aminoethyl)piperazine, the crude product was washed with diethylether affording the pure title compound as a yellow solid (η = 35%). ^1^H NMR (300 MHz, MeOD–*d*_4_), δ (ppm): 2.384−2.640 (m, 8H, piperazine), 2.675 (t, 2H, *J* = 6.0 Hz, PhthCH_2_*CH_2_*N), 3.52 (s, 2H, N*CH_2_*Ph) 3.801 (t, 2H, *J* = 6.0 Hz, Phth-*CH_2_*CH_2_N), 7.25−7.36 (m, 5H, *Ph*-CH_2_), 7.80−7.90 (m, 4H, *Phth*). M/z (ESI-MS): 350 (M+H)^+^.

*2-((1-Benzylpiperidine-3-yl)methyl)isoindoline-1,3-dione* (**3a**)**.** Starting from 3-(aminomethyl)piperidine and afterwards benzyl bromide, the crude solid compound was purified by chromatography column (with eluent ACN/H_2_O, 7/1) affording the pure title compound as yellow solid (η = 40%). ^1^H RMN (300 MHz, MeOD-*d*_4_), δ (ppm): 1.41–1.46, 1.71–1.99, 2.20–2.27 (m, 9H, piperidine), 2.88 (d, 2H, *J* = 12.0 Hz, N*CH_2_*CH), 3.49 (s, 2H, N*CH_2_*Ph), 7.40–7.57 (m, 9H, *Ph*CH_2,_ *Phth*). M/z (ESI-MS): 335 (M + 1)^+^.

*2-((1-Benzylpiperidine-2-yl)methyl)isoindoline-1,3-dione* (**4a**)**.** Starting from 2-(aminomethyl)piperidine and afterwards benzyl bromide, the crude solid compound was purified by recrystallization ACN/MeOH, affording a pure compound as a yellow solid (η = 43%, M.P. = 130 °C). ^1^H RMN (300 MHz, MeOD-*d*_4_), δ (ppm): 1.37−1.80 (M, 6H, piperidine), 2.02 (t, 2H, *J* = 12.0 Hz, piperidine, 2.91 (m, 1H, piperidine), 3.51 (s, 2H, *CH_2_*Ph), 3.58 (d, 2H, *J* = 12.0 Hz, N*CH_2_*CH), 7.34−7.29 (m, 5H, Ph), 7.89−7.79 (m, 4H, Phth); M/z (ESI-MS): 335 (M + 1)^+^.

*2-(2-(4-(2-Fluorobenzyl)piperazin-1-yl)ethyl)isoindoline-1,3-dione* (**10a**)**.** Starting from 1-(2-aminoethyl)piperazine and afterwards 2-fluorobenzyl-bromide, the crude solid compound was purified by washing with methanol, affording a pure compound as a beige solid (η = 25%, M.P. = 93−95 °C). H^1^ NMR (300 MHz, DMSO-*d*_6_), *δ* (ppm): 2.21−2.50 (m, 10H, piperazine, CH_2_N), 3.68 (t, 2H, *J* = 6 Hz, *CH_2_*NPhth), 7.18−7.11, 7.40−7.27 (2 × m, 4H, Ph), 7.82–7.88 (m, 4H, Phth). M/z (ESI-MS): 368.36 (M + 1)^+^. 

*2-(2-(4-(2-Nitrobenzyl)piperazin-1-yl)ethyl)isoindoline-1,3-dione* (**11a**)**.** Starting from 1-(2-aminoethyl)piperazine and afterwards 2-nitrobenzyl-bromide, the crude has been purified by washing with methanol affording a pure compound as a light yellow solid (η = 28%, M.P. = 100−103 °C). H^1^ NMR (300 MHz, MeOD-*d*_4_), *δ* (ppm): 2.26−2.58 (m, 8H, H8–11), 2.63 (t, 2H, *J* = 8 Hz, CH_2_N), 3.74 (s, 2H, N*CH_2_*Ph), 3.80 (t, 2H, *J* = 8 Hz, *CH_2_*NPhth), 7.48−7.42, 7.56–7.61 (2 × m, 4H,Ph), 7.78−7.86 (m, 4H, Phth). M/z (ESI-MS): 395.38 (M + 1)^+^.

*2-(2-(4-(Pyridin-2-ylmethyl)piperazin-1-yl)ethyl)isoindoline-1,3-dione* (**12a**)**.** Starting from 1-(2-aminoethyl)piperazine and afterwards 2-(bromomethyl)pyridine-HBr, the crude has been purified by washing with ether, affording a pure compound as a beige solid (η = 20%, M.P. = 93−95 °C). H^1^ NMR (400 MHz, MeOD–*d*_4_) *δ* (ppm): 2. 50–2.62 (m, 8H, piperazine), 2.66 (t, 2H, *J* = 8 Hz, *CH_2_*N), 3.63 (s, 2H, *CH_2_*Ph). 3.82 (t, 2H, *J* = 8 Hz, *CH_2_*NPhth), 7.28−7.33 (m, 1H, H2-py), 7.51 (d, 1H, *J* = 4 Hz, H4-Py), 7.86−7.78 (m, 1+4 H, H3-Py + Phth), 8.66 (d, 1H, *J* = 4 Hz, H1-Py). M/z (ESI-MS): 351.36 (M + 1)^+^.

##### 3.2.1.2. Deprotection of Primary Amine from the Isoindoline-1,3-Diones (**1b**−**4b**,**10b**−**12b**)

###### General Procedure

The benzylpiperidine- or benzylpiperazine- or methylpyridinepiperazine-isoindoline-1,3-dione derivatives (**1a**−**4a**; **10a**−**12a**) (1 eq) and hydrazine monohydrate (6 eq) were dissolved in absolute ethanol and warmed at 60 °C for 3 h. The reaction mixture was then filtered and the liquid filtrate was concentrated under reduced pressure. The residue was dissolved in ethyl acetate, washed with brine (three times), dried over anhydrous sodium sulphate, and concentrated under reduced pressure obtaining a yellow oil (η = 35–79%). 

*(1-Benzylpiperidin-4-yl)methanamine* (**1b**) Starting from **1a**, the pure compound was obtained as a yellow oil (η = 40%). ^1^H RMN (300 MHz, DMSO-*d*_6_), *δ* (ppm): 1.25−1.39, 1.58−1.69, 1.79−1.91 e 2.36−2.41 (m, 9H, piperidine), 2,77 (d, 2H, *J* = 12.0 Hz, NC*H_2_*CH), 3.42 (s, 2H, N*CH_2_*Ph), 7.19–7.36 (m, 5H, *Ph*-CH_2_). M/z (ESI-MS): 205.00 (M + 1)^+^.

*2-(4-Benzylpiperazin-1-yl)ethanamine* (**2b**)**.** Starting from **1a**, the pure compound was obtained as a yellow oil (η = 36%). ^1^H NMR (300 MHz, MeOD-*d*_4_), *δ* (ppm): 2.45 (t, 2H, *J* = 6 Hz, NH_2_CH_2_*CH_2_*N), 2.60–2.52 (m, 8H, piperazine), 2.75 (t, 2H, *J* = 6 Hz, NH_2_*CH_2_*CH_2_N), 3.53 (s, 2H, N*CH_2_*Ph), 7.21–7.37 (m, 5H, Ph). M/z (ESI-MS): 220 (M + 1)^+^.

*(1-Benzylpiperidin-3-yl)methanamine* (**3b**)**.** Starting from **3a**, the title pure product was obtained (η = 37%). ^1^H RMN (300 MHz, MeOD-*d*_4_), *δ* (ppm): 1.69−1.96, 2.32–2.39 (m, 7H, piperidine), 2.54 (t, 2H, *J* = 12 Hz, piperidine), 2.97 (d, 2H, *J* = 9 Hz, N*CH_2_*CH), 3.56 (s, 2H, N*CH_2_*Ph), 7.81−7.90 (m, 5H, Ph). M/z (ESI-MS): 205 (M + 1)^+^.

*(1-Benzylpiperidin-2-yl)methanamine* (**4b**)**.** Starting from **4a**, the pure title product was obtained (η = 35%). ^1^H RMN (300 MHz, MeOD-*d*_4_), *δ* (ppm): 1.24−1.86 (2 × m, 6H, piperidina), 2.40 (t, 2H, *J* = 12.0 Hz piperidina), 2.78 (m, 1H, *J* = 12.0 Hz, NC*H2*CH), 2.91 (m, 1H, piperidina), 3.51 (s, 2H, NC*H2*Ph), 7.34−7.19 (m, 5H, *Ph*). M/z (ESI-MS): 205 (M + 1)^+^.

*2-(4-(2-Fluorobenzyl)piperazin-1-yl)ethanamine* (**10b**)**.** Starting from **10a**, η = 73%. ^1^H NMR (400 MHz, MeOD-*d*_4_), *δ* (ppm): 2.46 (t, 2H, *J* = 8 Hz, N*CH_2_*CH_2_NH_2_), 2.69–2.48 (m, 8H, piperazine), 2.74–2.78 (t, 2H, *J* = 8 Hz, NCH_2_*CH_2_*NH_2_), 3.62 (s, 2H, NC*H_2_*Ph), 7. 06−7.10, 7.13−7.17, 7.28−7.33, 7.38−7.42 (4 × m, 4H, Ph). M/z (ESI-MS): 238.22 (M + 1)^+^.

*2-(4-(2-Nitrobenzyl)piperazin-1-yl)ethanamine* (**11b**)**.** Starting from **11**, η = 79%. ^1^H NMR (300 MHz, MeOD-*d*_4_), *δ* (ppm): 2.41−2.55 (m, 10H, Pyridine+ NC*H_2_*CH_2_NH_2_), 2.74 (t, 2H, *J* = 6 Hz, NCH_2_*CH_2_*NH_2_), 3.79 (s, 2H, *CH_2_*Ph), 7.44−7.50, 7.58−7.60 (m, 3H, Ph), 7.81 (d, 1H, *J* = 9 Hz, Ph). M/z (ESI-MS): 265 (M + 1)^+^. 

*2-(4-(Pyridin-2-ylmethyl)piperazin-1-yl)ethanamine* (**12b**)**.** Starting from **12a**, η = 36.3%. ^1^H NMR (300 MHz, MeOD-*d*_4_), *δ* (ppm): 2.45 (t, 2H, *J* = 6 Hz, N*CH_2_*CH_2_NH_2_) 2.64−2.55 (m, 8H, piperazine), 2.74 (t, 2H, *J* = 6 Hz, NCH_2_*CH_2_*NH_2_), 3.67 (s, 2H, *CH_2_*Py), 7.30−7.34 (m, 1H, Py-H-2), 7.54 (d, 1H, *J* = 6 Hz, Py-H-4), 7.80−7.85 (m, 1H, H3Py), 8.48 (d, 1H, *J* = 6 Hz, Py-H-1). M/z (ESI-MS): 221 (M + 1)^+^.

#### 3.2.2. Synthesis of 2-(X-2-hydroxyphenyl)-1*H*-benzo[*d*]imidazole-5-carboxylic Acids (X = 5-F, 5-NO_2_, 4-OMe)

##### General Procedure (Method B)

3,4-Diaminobenzoic acid (1 eq), a suitable salicylaldehyde (1 eq) and Na_2_S_2_O_5_ (1.4 eq) were dissolved in choline chloride/glycerol (1:2), mol/mol) and warmed at 50 °C for 30 min. Then the reaction mixture was cooled at rt, added to water and filtrated. The solid obtained was washed with dichloromethane, affording beige-yellow solids with yield 67%−97%. 

*2-(5-Fluoro-2-hydroxyphenyl)-1*H*-benzo[*d*]imidazole-5-carboxylic acid* (**6c**)**.** Starting from 5-fluoro-salicylaldehyde, the title compound was obtained as a beige solid (η = 97%), M.P. = 250 °C. ^1^H NMR (300 MHz, DMSO-*d*_6_), *δ* (ppm): 7.08 (s, 1H, BIM-H-7) 7.26 (d, 1H, *J* = 6.0 Hz, BIM-H-4),7.74 (d, 1H, *J* = 6.0 Hz, BIM-H-5), 7.91 (d, 1H, *J* = 9.0 Hz, BIM-H-2), 8.00 (d, 1H, *J* = 9.0 Hz, BIM-H-3),8.25 (s, 1H, BIM-H-1), 12.78 (s, 1H, COOH). M/z (ESI-MS): 273 (M + 1)^+^. 

*2-(5-Nitro-2-hydroxyphenyl)-1*H*-benzo[*d*]imidazole-5-carboxylic acid* (**7c**)**.** Starting from 5-nitro-salicylaldehyde, the title compound was obtained as a beige solid (η = 78%), M.P. > 300 °C. ^1^H NMR (300 MHz, DMSO-*d*_6_), *δ* (ppm): 7.06−7.11 (m, 1H, BIM-H-2), 7.25–7.32 (m, 1H, BIM-H-4), 7.73−7.76 (d, 1H, *J* = 9.0 Hz, BIM-H-2), 7.89−7.96 (m, 2H, BIM-H-1, BIM-H-5), 8.24 (s, 1H, BIM-H-7), 12.78 (s, 1H, COOH). M/z (ESI-MS): 300 (M + 1)^+^. 

*2-(4-Metoxy-2-hydroxyphenyl)-1*H*-benzo[*d*]imidazole-5-carboxylic acid* (**9c**)**.** Starting from 4-metoxy-salicylaldehyde, the title compound was obtained as a beige solid (η = 67%), M.P. > 300 °C. ^1^H NMR (300 MHz, DMSO-*d*_6_), *δ* (ppm): 3.82 (sg, 3H, OCH_3_), 6.62 (s, 1H, BIM-H-4) 6.66 (d, 1H, *J* = 9.0 Hz, BIM-H-6), 7.68 (d, 1H, *J* =9.0 Hz, BIM-H-7), 7.88 (d, 1H, *J* = 9.0 Hz, BIM-H-2), 7.98 (d, 1H, *J* = 9.0 Hz, BIM-H-3), 8.18 (s, 1H, BIM-H-1), 12.96 (s, 1H, COOH). M/z (ESI-MS): 285 (M + 1)^+^. 

#### 3.2.3. Synthesis of the Final Conjugates (**1**–**12**)

##### General Procedure

To a solution of the carboxylic acid derivatives (**1c**, **5c**, **6c**, **7c**, **9c**) (1 eq) and the amine derivatives (**1**–**4b**, **10**–**12b**) (1 eq) in anhydrous DMF, was added *N*-hydroxysuccinimide (1 eq) and DCC (1 eq) and the reaction mixture was left stirring for 24 h at rt. Then, the precipitate obtained (urea) was filtered off and the liquid phase was diluted with ethyl acetate and washed with brine (three times). The combined organic phase was dried over anhydrous Na_2_SO_4_, concentrated under reduced pressure, and purified by recrystallisation in EtOAc or Et_2_O and ACN/MeOH, or column chromatography, affording the pure final conjugate as a solid.

*N-((1-Benzylpiperidin-3-yl)methyl)-2-(2-hydroxyphenyl)-1*H*-benzo[*d*]imidazole-6-carboxamide* (**3**)**.** Starting from **3b** and **1c**, the title compound was obtained as a beige solid (η = 22%), M.P. = 220−222 °C. ^1^H RMN (300 MHz, MeOD-*d*_4_), *δ* (ppm): 1.49−1.94 (m, 5H, piperidine), 2.64− 2.89 (m, 4H, N*CH_2_* (piperidine)), 3.18 (m, 2H, NH*CH_2_*, 3.53 (s, 2H, N*CH_2_*Ph): ^13^C RMN (75.5 MHz, MeOD-*d*_4_), *δ* (ppm): 25.41, 27.80, 29.32, 43.06, 56.94, 59.71, 64.82, 114.2, 116.4, 117.9, 118.4, 121.9, 122.4, 127.6, 128.2, 128.9, 129.8, 130.3, 131.8, 139.0, 145.3,152.7, 154.1, 166.9. M/z (ESI-MS): 441.36 (M + 1)^+^.

*N-((1-Benzylpiperidin-2-yl)methyl)-2-(2-hydroxyphenyl)-1*H*-benzo[*d*]imidazole-6-carboxamide* (**4**). Starting from **4b** and **1c**, the title compound was obtained as a beige solid (η = 25%), M. P. = 226−227 °C. ^1^H NMR (300 MHz, DMSO-*d*_6_), *δ* (ppm): 1.24−1.71 (m, 6H, Pip), 2.63–2.73 (m, 3H, Pip), 3.42 (d, 2H, *J* = 12 Hz, CH*CH_2_*N), 3.64 (s, 2H, *CH_2_*Ph), 4.05 (sb, 1H, NH), 7.05−7.08 (m, 2H, H24, H26), 7.23−7.45 (m, 6H, H1 a H5 e H25), 7.65 (d, 1H, *J* = 6 Hz, H27), 7.80 (d, 1H, *J* = 6 Hz, H18), 8.09 (d, 1H, *J* = 9 Hz, H19), 8.26 (s, 1H, H16), 8.41 (s, 1H, NH). ^13^C NMR (75.5 MHz, DMSO-d_6_), *δ* (ppm): 22.75, 24.38, 28.96, 33.96, 50.01 50.58, 60.08, 111.56, 112.94, 117.36, 117.73, 119.74, 122.14, 123.43, 127.00, 128.57, 128.97, 132.60, 135.69, 140.15, 141.03, 153.79, 158.47, 166.91. M/z (ESI-MS): 441.24 (M + 1) ^+^. 

*N-((1-Benzylpiperidin-4-yl)methyl)-2-(2-hydroxy-5-fluorophenyl)-1*H*-benzo[*d*]imidazole-5-carboxamide* (**6**). Starting from **1b** and **6c**, the title compound was obtained as a white solid (η = 24%) M.P. = 241−243 °C. ^1^H NMR (300 MHz, MeOD-*d*_4_), *δ* (ppm): 1.01−1.24 (m, 4H, piperidine), 163−1.91 (m, 5H, piperidine), 2.81 (d, 2H, *J* = 12.0 Hz, NC*H2*CH), 3.49 (s, 2H, NC*H2*Ph), 5.59 (b, 1H, *NH*), 7.12−7.03 (m, 2H, BIM-H-5,6) 7.19−7.39 (m, 5H, *Ph*-CH2), 7.69 (s, 1H, BIM-H-7), 7.81 (d, 1H, *J* = 8.0 Hz, BIM-H-4), 7.93 (d, 1H, *J* = 8.0 Hz, BIM-H-3), 8.51 (s, 1H, BIM-H-2). ^13^C NMR (100 MHz, MeOD-*d*_4_), *δ* (ppm): 27.3, 37.2, 43.2, 53.1, 63.3, 115.2, 116.5, 117.6, 119.9, 122.3, 126.6, 128.2, 128.9, 132.3, 138.4, 146.2, 149.3, 151.3, 152.7, 166.8. M/z (ESI-MS): 459.23 (M + 1)^+^.

*N-(2-(4-Benzylpiperazin-1-yl)ethyl)-2-(2-hydroxy-5-nitrophenyl)-1*H*-benzo[*d*]imidazole-5-carboxamide* (**7**). Starting from **2b** and **7c**, with final purification through column chromatography (eluent: DCM/MeOH 98:2) afforded a yellow solid (η = 28%), M.P. = 225–227 °C. ^1^H NMR (300 MHz, MeOD-*d*_4_), *δ* (ppm): 2.77–2.94 (m, 10H, 8 piperazine + 2 NH_2_CH_2_*CH_2_*N), 3.66–3.75 (m, 4H, N*CH_2_*Ph and NH_2_*CH_2_*CH_2_N), 7.07–7.38, 7.77–7.83, and 8.18–9.05 (3 × m, 11H, Ar-H). ^13^C NMR (100 MHz, MeOD-*d*_4_), *δ* (ppm): 30.3, 36.2, 45.7, 53.6, 62.8, 113.3, 117.8, 119.5, 127.0, 127.3, 128.6, 129.3, 129.8, 132.5, 139.1, 143.9, 157.1, 160.8, 167.9. M/z (ESI-MS): 501.24 (M + 1)^+^.

*N-(2-(4-Benzylpiperazin-1-yl)ethyl)-2-(2-hydroxy-5-fluorophenyl)-1*H*-benzo[*d*]imidazole-5-carboxamide* (**8**). Starting from **2b** and **6c**, with final recrystallization from ACN/MeOH, afforded the title compound as a white-beige solid (η = 27%) M.P. = 246−248 °C. ^1^H NMR (300 MHz, MeOD-*d*_4_), *δ* (ppm): 2.77–2.94 (m, 10H, piperazine + 2 NH_2_CH_2_*CH_2_*N), 3.66–3.75 (m, 4H, 2 N*CH_2_*Ph + 2 NH_2_*CH_2_*CH_2_N), 7.03–7.20, 7.34–7.37, and 7.69–8.16 (m, 11H, Ar-H). ^13^C NMR (100 MHz, MeOD-*d*_4_), *δ* (ppm): 30.3, 36.2, 45.7, 53.6, 62.8, 113.3, 117.8, 119.5, 127.0, 127.3, 128.6, 129.3, 129.8, 132.5, 139.1, 154.6, 157.1, 160.8, 167.9. M/z (ESI-MS): 474.22 (M + 1)^+^.

*N-(2-(4-Benzylpiperazin-1-yl)ethyl)-2-(4-metoxy-2-hydroxyphenyl)-1*H*-benzo[*d*]imidazole-5-carboxamide* (**9**). Starting from **2b** and **9c**, with final recrystallization from ACN/MeOH afforded the pure title compound as white solid (η = 32%), M.P. = 249−251 °C. ^1^H NMR (300 MHz, MeOD-*d*_4_), *δ* (ppm): 2.77–2.94 (m, 10H, piperazine + NH_2_CH_2_*CH_2_*N), 3.66–3.75 (m, 4H, 2 N*CH_2_*Ph + 2 NH_2_*CH_2_*CH_2_N), 3.87 (s, 3H, OCH_3_), 6.70–6.74, 7.29–7.36, and 7.61–8.10 (3 × m, 11H, Ar-H). ^13^C NMR (100 MHz, MeOD-*d*_4_), *δ* (ppm): 30.3, 36.2, 45.7, 53.6, 62.8, 113.3, 117.8, 119.5, 127.0, 127.3, 128.6, 129.3, 129.8, 132.5, 139.1, 157.1, 160.8, 162.1, 167.9. M/z (ESI-MS): 486.25 (M + 1)^+^. 

*N-(2-(4-(2-Fluorobenzyl)piperazin-1-yl)ethyl)-2-(2-hydroxyphenyl)-1*H*-benzo[*d*]imidazole-6-carboxamide* (**10**). Starting from **10b** and **1c**, the pure product was purified by column chromatography (eluent: ACN/H_2_O, 9:1) (η = 47%), M.P. = 219−222 °C. ^1^H NMR (400 MHz, MeOD–*d*_4_), *δ* (ppm): 2.63−2.90 (m, 10H, piperazine + N*CH_2_*CH_2_NH), 3.62 (t, 2H, *J* = 6 Hz, NCH_2_*CH_2_*NH), 3.72 (s, 2H, N*CH_2_*Ph), 6.98 -7.20 (m, 4H, H4-5, H24, H26), 7.30−7.44 (m, 3H, H1-2, H14), 7.67 (d, 1H, *J* = 9 Hz, H27), 7.78 (d, 1H, *J* = 6 Hz, H16), 7.97 (d, 1H, *J* = 9 Hz, H17), 8.14 (s, 1H, H20). ^13^C NMR (100 MHz MeOD–*d*_4_), *δ* ppm: 36.26, 51.65, 52.30, 54.32, 56.78, 112.62, 114.87, 115.05, 117.34, 119.41, 123.88, 126.32, 131.88, 132.12, 158.18. M/z (ESI-MS): 474.31 (M + 1)^+^.

*2-(2-Hydroxyphenyl)-N-(2-(4-(2-nitrobenzyl)piperazin-1-yl)ethyl)-1*H*-benzo[*d*]imidazole-6-carboxamide* (**11**). Starting from **11b** and **1c** the pure title product was purified by column chromatography (eluent: AcOEt/MeOH, 9:1), (η = 40%), M.P. = 242−244 °C. ^1^H NMR (400 MHz, DMSO–*d*_6_), *δ* (ppm): 2.36−2.53 (m, 10H, piperazine + N*CH_2_*CH_2_NH) 3.63−3.74 (m, 4H, N*CH_2_*Ph + NCH_2_*CH_2_*NH), 5.51 (s, 1H, OH), 8.39 (s, 1H, H20), 7.01−7.06 (m, 2H, H24 and H26), 7.39−7.42 (m, 1H, H25), 7.50−7.53 (m, 1H, H27), 7.60−7.70 (m, 3H, H3, H4 and H5), 7.78 (d, 2H, *J* = 8 Hz, H2), 7.85 (d, 2H, *J* = 8 Hz, H16), 8.08 (d, 2H, *J* = 8 Hz, H17), 8.14 (s, 1H, C-CH-C). ^13^C NMR (100 MHz, DMSO–*d*_6_), *δ* ppm: 34.36, 35.31, 42.99, 46.81, 62.49, 66.87, 68.25, 122.48, 123.62, 127.31, 129.13, 134.07, 136.31, 138.42, 140.98, 142.11, 142.38, 142.85, 157.52, 157.80, 159.61, 166.38, 167.80, 176.01. M/z (ESI-MS): 501.29 (M + 1)^+^.

*2-(2-Hydroxyphenyl)-N-(2-(4-(pyridin-2-ylmethyl)piperazin-1-yl)ethyl)-1*H*-benzo[*d*]imidazole-6-carboxamide* (**12**). Starting from **12b** and **1c** the title product was purified by recrystallization from ACN/MeOH, (η = 41%), M.P. = 220−222 °C. ^1^H NMR (300 MHz, MeOD–*d*_4_), *δ* (ppm): 2.50−2.62 (m, 8H, piperazine), 2.66 (t, 2H, *J* = 6Hz, H11), 3.58 (t, 2H, *J* = 6 Hz, H12), 3.68 (s, 2H, N*CH_2_*Ph), 6.97−7.05 (m, 2H, H23 and H25), 7.3−7.4 (m, 2H, H24 and H2), 7.54 (d, 1H, *J* = 9 Hz, H4), 7.66 (d, 1H, *J* = 9 Hz, H26), 7.76−7.85 (m, 2H, H3 and H17), 7.96 (d, 1H, *J* = 9 Hz, H18), 8.13 (s, 1H, H1), 8.47 (d, 1H, *J* = 6 Hz, H1). ^13^C NMR (100 MHz MeOD–*d*_4_), *δ* ppm: 36.49, 52.41, 52.73, 56.96, 63.20, 112.68, 116.80, 119.12, 121.93, 122.70, 123.71, 126.29, 128.83, 131.85, 137.11, 148.25, 149.64, 153.83, 155.50, 157.68, 158.44, 161.24, 169.17. M/z (ESI-MS): 457.31 (M + 1)^+^.

### 3.3. Molecular Modeling: Docking and Pharmacokinetics Studies

Docking simulations were carried out using the Gold v. 5.1 package of software [46]. The model structure for the active site of AChE was retrieved from the Brookhaven protein database (RCSB) (PDB, entry 1 EVE) [24], in particular, from the X-ray crystal structure of *Torpedo Californica* AChE (*Tc*AChE) complexed with the cholinesterase inhibitor drug in clinical use (donepezil, DNP), hereinafter named as the original ligand. In spite of some known differences between the inhibitor-enzyme complexes, reported for the human acetylcholine esterase, *h*AChE, and the electric ray homologue (*Torpedo californica*, *Tc*AChE) both enzymes are quite conservative in terms of the main amino acid residues that line up the active site gorge [47], and so this docking study was performed with the *Tc*AChE molecular model. 

For the simulations, the original complex of *Tc*AChE-DNP, retrieved from RCSB, was treated with MAESTRO v. 9.3 [48] to get the model structure of the receptor enzyme, by removing the original ligand (DNP), solvent and co-crystallization molecules, and subsequently adding hydrogen atoms. This program was also used to build the ligand structures which were then optimized (1000 cycles of random conformational search and 2500 optimization steps) with the program Ghemical v 2.0 [49,50]. The ligands were subsequently docked into the AChE active site, using GOLD program with the corresponding default parameters (except “allow early termination” option) and the Astex Statistical Potential (ASP) scoring function. The docking site was defined by establishing a box within 10 Å from the original ligand in the above mentioned PDB structure. The first-ranked structures obtained from these calculations were then compared with the original ligand and analyzed in their interactions with the active site residues. The results were visualized using Chimera 1.6 [51].

Moreover, pharmacokinetic molecular descriptors were *in silico* evaluated. Parameters such as the octanol-water partition coefficient (clog *P*), blood–brain barrier partition coefficient (log BB), the ability to be absorbed through the intestinal tract (Caco-2 cell permeability), and CNS activity were calculated. The chemical structures were previously minimized in Maestro v. 9.3 [48] and then they were submitted to the calculation of these relevant pharmacokinetic properties and descriptors using QikProp v. 2.5 [43].

### 3.4. Bio-Analytical Procedures

#### 3.4.1. Inhibition of Acetylcholinesterase

An adaptation of the Ellman´s method, previously described, was used to measure the acetylcholinesterase inhibitory activity [52,53]. The assay solution contained 374 µL of 4-(2-hydroxyethyl)-1-piperazineethanesulfonic acid (HEPES) buffer (50 mM, pH 8.0), 476 µL of 5,5’-dithiobis-(2-nitrobenzoic acid) (DTNB, 3 mM), a variable volume (10–50 µL) of the stock solution of each compound in methanol (1 mg/mL), 25 µL of AChE (type VI-S, from electric eel) stock solution, and the necessary amount of methanol to obtain 0.925 mL of sample mixture in a 1 mL cuvette. The samples were incubated for 15 min and then 75 µL of acetylthiocholine iodide (AChI) solution (16 mM) was added. The reaction was monitored for 5 min at 405 nm. Assays were run with a blank containing all the components except AChE, which was replaced by HEPES buffer. The rates of the reaction were calculated as well as the enzyme activity. A control reaction was carried out using methanol as a sample solution and it was considered as 100% activity. The percentage inhibition of the enzyme activity due to the presence of increasing test compound concentration was calculated by the following Equation (1),

I% = 100 − 100 × *v*_i_/(*v*_i_ − *v*_o_) (1)

where *v*_i_ = starting reaction rate in the presence of inhibitor; *v*_o_ = starting reaction rate of the control reaction.

The inhibition curves were obtained by plotting the percentage of enzymatic inhibition versus inhibitor concentration and a calibration curve was got from which the linear regression parameters were obtained.

#### 3.4.2. Inhibition of Self- and Cu-Mediated Aβ_1–42_ Aggregation 

The Aβ peptide was treated with 1,1,1,3,3,3-hexafluoropropan-2-ol (HFIP) and dissolved in a CH_3_CN/Na_2_CO_3_ (300 μM)/NaOH (250 μM) (48.3:48.3:4.3, *v*/*v*/*v*) mixture in order to have a stable stock solution, avoiding self-aggregation of Aβ_1–42_. Then, the prepared 500 μM solution was diluted at 40 μM in phosphate buffer (0.215 M, pH 8.0). 

The solutions of the tested compounds were prepared in MeOH (1 mg/mL), being further diluted in phosphate buffer to a concentration of 480 μM. For copper-induced aggregation studies, a solution of CuCl_2_ 240 μM was prepared from a stock standard solution (0.015 M). To perform the assays for the Aβ_1–42_ aggregation inhibition, a reported method, based on the fluorescence emission of thioflavin T (ThT), was followed [36,37,54]. Aβ_1–42_ (40 μM) was incubated at 37 °C, for 24 h with or without Cu(II) (40 μM) in a phosphate buffer in presence or absence of the single ligand (40 μM and 20 μM for compound **4**). Then, the samples were added to a 96-well plate with 180 μM of 5 μM ThT in 50 mM glycine-NaOH (pH 8.5) buffer. Blank samples were prepared without the peptide for each concentration. The ThT fluorescence was measured at 446 nm (excitation) and 485 nm (emission). The inhibition percentage of aggregation was calculated by Equation (2), in which IF_i_ and IF_0_ corresponded to the fluorescence intensities, in the presence and in the absence of the tested compound, subtracted of the fluorescence intensities due to the respective blanks.

I% = 100 − (IF_i_/IF_0_ × 100) (2)

The reported values were obtained as the mean ± SEM of duplicate of three different experiments.

#### 3.4.3. Metal Chelation

Solutions: the aqueous copper (CuCl_2_, 0.015 M) and zinc (ZnCl_2_, 0.0156 M) stock solutions for potentiometric equilibrium studies were prepared from 1000 ppm standards (Titrisol) and their metal content was evaluated by atomic absorption. The titrant solution (0.1 M KOH) was obtained from a carbonate-free commercial concentrate (Titrisol) and standardized by potentiometric titration with potassium hydrogen phthalate. The titrant solution was discarded whenever the percentage of carbonate (Gran’s method) [55] was about 0.5% of the total amount of base. 

Measurements**:** compounds **7** and **12** were titrated in 50% (*w*/*w*) DMSO/H_2_O medium, at *T* = 25.0 ± 0.1 °C and ionic strength (*I*) 0.1 M KCl, using 0.1 M KOH as titrant. Both glass and Ag/AgCl reference electrodes were previously conditioned in DMSO/H_2_O mixtures with increasing amounts of DMSO and the response of the glass electrode was controlled by strong acid–strong base (HCl/KOH) calibrations with the determination of the Nernst parameters by Gran’s method [55]. The measurements were performed in a final volume of 30.00 mL, with a ligand concentration (*C*_L_) of 6.7 × 10^−4^ M, under different *C*_M_/*C*_L_ ratios: 0:1 (L), 1:1, and 1:2 (M = Cu, Zn). All titrations were performed in duplicate and under the stated experimental conditions the p*K*_w_ value (13.6) was determined and subsequently used in the computations. The stepwise protonation constant of the ligand, *K_i_* = [H_i_L]/([H_i–1_L][H]), and the overall metal–complex stability constants, $\beta_{M_{m}H_{h}L_{l}}$ = [M_m_H_h_L_l_]/([M]^m^[H]^h^[L]^l^), were calculated by fitting the potentiometric titration data with Hyperquad 2008 [26]. The metal hydrolysis constants were determined under the defined experimental conditions (*I* = 0.1 M KCl, 50% *w*/*w* DMSO/H_2_O, T = 25.0 ± 0.1 °C) and the following values of stability constants were included in the fitting of experimental data towards the equilibrium models related to the Cu^2+^/L and Zn^2+^/L systems: log$\beta_{Cu_{2}H_{-2}}$ = −9.99; log$\beta_{ZnH_{-2}}$ = −14.79, log$\beta_{ZnH_{-3}}$ = −23.48. Species distribution curves were plotted with the Hyss program [26].

#### 3.4.4. Cell Viability and Neuroprotection 

SH-SY5Y human neuroblastoma cell line (ATCC-CRL-2266) was cultured in Dulbecco’s Modified Eagle Medium (DMEM) (Gibco-Invitrogen, Life Technologies Ltd., Liverpool, UK) supplemented with 10% of heat inactivated fetal bovine serum, 50 U/mL penicillin, and 50 μg/mL streptomycin, at 37 °C in 5% CO_2_. For the cell viability experiments, cells were plated with a density of 0.12 × 10^6^ cells/mL. All the tested compounds (from **7** to **12**) were dissolved in DMSO and aliquots were stored at −20 °C with a stock concentration of 25 mM. To choose the highest non-toxic concentration of the compounds, a concentration screening was performed (from 2.5 μM to 10 μM). For the experiments, all the compounds were added to the medium at a final concentration of 2.5 μM. The concentration of DMSO in culture media did not exceed 0.05% (*v*/*v*) and no alterations were detected in the SH-SY5Y cell line. Cells were pre-incubated for 1 h with the compounds and then incubated with Aβ_1–42_ or with Fe/Asc for an additional 24 h. Aβ_1–42_ (Bachem, Torrance, CA, USA) stock with a concentration of 276.9 μM was prepared in sterile water and a final concentration of 1 μM was added to the culture medium. Both stock solutions of ferrous sulfate (Fe) and *L*-Ascorbic acid (Asc) (Sigma Chemical Co, St. Louis, MO, USA) were prepared in sterile water and 2.5 mM of ferrous sulfate and 5 mM of L-Ascorbic acid were added together to the culture medium. For all the experiments, control conditions were performed in which the compounds were tested and Aβ_1–42_ or Fe/Asc were not added.

Cell viability was determined using the colorimetric 3-(4,5-dimethylthiazol-2-yl)-2,5-diphenyltetrazolium bromide (MTT) assay [56]. In viable cells, cellular dehydrogenases metabolize the MTT into a formazan that absorbs light at 570 nm. After the incubations, the culture medium was aspirated and 0.3 mL of MTT (0.5 mg/mL) was added to each well. After incubation at 37 °C for 3 h, formazan precipitates were solubilized with 0.3 mL of acidic/isopropanol (0.04 M HCl/Isopropanol). The absorbance of the plate was measured at 570 nm. Cell reduction ability was normalized to untreated control cells. 

All data were expressed as mean ± SEM of at least four independent experiments performed in duplicates. Statistical analyses were performed using one-way ANOVA followed by Bonferroni multiple comparisons procedure post hoc test. *P* value < 0.05 was considered statistically significant.

## 4. Conclusions

Due to the complexity of AD pathogenesis and concomitant absence of cure, we have been engaged in the search for molecular entities capable of hitting multiple targets of this disease and thus being possibly explored as chemical tools against AD. The present strategy has been focused on the repositioning of approved drugs based on AChE inhibition, namely donepezil (DNP). Therefore, the MTDLs developed herein integrate mimetics of the active molecular fragment of DNP merged with other moieties, in order to combine the symptomatic amelioration by AChEi with other disease-modifying roles involved in amyloid plaque formation, namely inhibition of Aβ aggregation, as well as modulation of metal dyshomeostasis and oxidative stress. In particular, challenged by previous results with simple (DNP-BIM) conjugates (DNP mimetics coupled with a 2-hydroxyphenylbenzimidazole (BIM)), the present work exploited the effect on the activity of some structural modifications, namely by positional isomerization and substituent introduction. Regarding the positional isomers (**3**, **4**, **5**) of compound **1**, there is a general slight decrease of the AChEi activity, namely for compound **4**, due to some apparent structural distortion, although they keep identical capacity to inhibit Aβ aggregation. All the compounds are able to chelate metal ions through their BIM moieties and, remarkably, isomer **5**, due to the closeness of the carbonyl to the bidentate BIM unity provides a three-dentate chelating moiety, having higher capacity for copper chelation. This may be responsible for the more than 50% increase on Cu-induced Aβ aggregation relative to the self-induced one observed for **5**. Concerning the effect of the substituents on the cholinergic activity, the best results were obtained for the conjugates with the fluorine atom in each moiety (**6**,**8** and **10**) and also with the metoxyl group in the benzyl ring (**9**), while lowest activity was revealed by the compounds with the nitro substituents (**7**,**11**), but the worst results were obtained for the pyridinium derivative (**12**). Regarding the effect of a selection of DNP-BIM hybrids in neuroblastoma cells, compounds **9** and **10** showed a remarkable reduction of Aβ-induced cell toxicity, although none of the tested compounds presented a statistically significant neuroprotective effect by preventing ROS production. Overall, among these DNP-BIM hybrids, structural isomerization did not bring general improvement of properties; regarding the substituent modification, excluding the nitro-substituted and pyridinium derivatives, a set of substituted conjugates, namely methoxy and fluorine substituent groups, showed very good properties and so they deserve to be further explored in the development of new hybrids aimed at the discovery of novel anti-AD drugs. 

## Figures and Tables

**Table 1:** Stepwise protonation constants (log *K*_i_) of compounds **7**, **12** and other analogues for comparison purposes, as well as global formation constants ^a^ for their Cu(II) and Zn(II)) complexes (*T* = 25.0 ± 0.1 ^°^C, *I* = 0.1 M KCl, 50% *w*/*w* DMSO/water) and pM ^b^ values.

Compound	M_m_H_h_L_l_ _(mhl)_	log *K*_i_	$\log\;\beta_{{\mathrm{Cu}}_{m}H_{h}L_{l}}$	$\log\;\beta_{{\mathrm{Zn}}_{m}H_{h}L_{l}}$
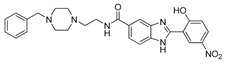 **7**	(011) (021) (031) (111) (101) (122) (102) (1–12) **pM**	6.99(2) 5.51(5) 2.95(6)	14.23(5) - 26.90(7) 15.27(8) 7.30(9) 10.9	10.40(8) 4.98(8) 21.92(7) 10.22(8) - 6.5
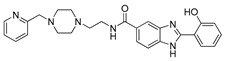 **12**	(011) (021) (031) (041) (111) (101) (102) (1–12) **pM**	9.09(3) 6.44(4) 3.64(5) 3.17(6)	16.26(3) 11.18(5) 17.14(7) 7.88(7) 10.4	12.58(4) 6.26(5) 10.54(5) 2.07(7) 6.3
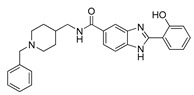 **1** ^c^	(011) (021) (031) **pM**	8.671(1) ^c^ 7.39(1) ^c^ 3.21(1) ^c^	10.7/	6.3
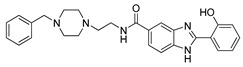 **2** ^c^	(011) (021) (031) **pM**	9.04(3) 6.76(6) 3.37(7)	10.7	6.3
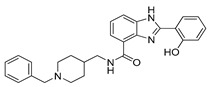 **5** ^d^	(011) (021) (031) **pM**	8.98(2) 7.78(4) 2.86(5)	14.3	6.4

^a^$\beta_{M_{m}H_{h}L_{l}}$ = [M_m_H_h_L_l_]/([M]^m^[H]^h^[L]^l^); ^b^ pM = −log[M] at pH 7.4 (*C*_L_/*C*_M_ = 10, *C*_M_ = 10^−6^ M), ref [32]; ^c^ ref. [33]; ^d^ ref [31].

**Table 2:** Summary of results obtained for inhibition of AChE and β–amyloid (Aβ) aggregation of the DNP-BIM hybrids.

Compound	*Tc*AChE Inhibition ^a^ IC_50_ (μM)	Aβ_1–42_ Aggregation Inhibition^b,c^ (%)	
		Self-Induced	Cu-Induced
**1**	4.2 ± 0.8 ^d^	48.8 ^e^	69.0 ^e^
**2**	6.9 ± 0.6 ^d^	25.3 ^d^	37.7 ^d^
**3**	5.0 ± 0.5	71.4	81.8
**4**	14 ± 1	47.6	55.9
**5**	9 ± 1 ^e^	38.6 ^e^	58.9 ^e^
**6**	1.67 ± 0.05	51.2	61.0
**7**	21 ± 3	57.6	71.4
**8**	4.3 ± 0.3	50.6	55.2
**9**	1.87 ± 0.8	43.7	66.0
**10**	4.32 ± 0.03	39.1	75.8
**11**	20 ± 1	22.7	43.5
**12**	>10^2^	30.1	44.4
**DNP** ^f^	0.026	-	-

^a^ The values are means of three independent experiments ± SD; AChE from *Electric eel*. ^b^ Inhibition of self-mediated Aβ_42_ aggregation (%) with or without copper (40 µM). The thioflavin-T fluorescence method was used, and the measurements were carried out in the presence of an inhibitor (40 µM and 20 µM for compound **4**). ^c^ The values are the means of three independent measurements in duplicate (SEM < 10%). ^d^ Reference [31] (A*β* aggregation inhibitory assays for *C*_inhibitor_ = 80 µM); ^e^ reference [31]; ^f^ reference [35].

**Table 3:** Pharmacokinetic properties of the compounds as predicted *in silico* by software QikProp v.2.5 [43].

Comp.	MW (Da)	*c*log *P*	log BB	Caco-2 Permeability (nm/s)	CNS	Violations of Lipinski’s Rule
1	440.54	4.386	−0.730	194	-	0
2	455.55	2.587	−0.097	48	+/−	0
3	440.54	4.122	−0.825	155	-	0
4	440.54	4.202	−0.981	121	-	0
5	440.54	4.352	−0.638	225	+/−	0
6	458.53	4.513	−0.663	177	+/-	0
7	516.55	2.421	−1.148	7	-	1
8	473.54	3.490	−0.147	61	+/−	0
9	485.58	2.840	−0.029	79	-	0
10	473.54	3.455-	−0.385	45	+/−	0
11	500.55	2.604	−1.641	6	-	1
12	456.54	2.686	−0.597	34	+/−	0
DNP	379.50	4.269	0.132	893	+	0

## Supplementary Materials

The following are available online, Figure S1: Redocking of original ligand (DNP) from PDB code 1EVE [1], inside the TcAChE active site, under the same simulation conditions used for the docking of the ligands in the present study.
