# Supplementary material for: Design, Synthesis, and In Vitro Evaluation of Hydroxybenzimidazole-Donepezil Analogues as Multitarget-Directed Ligands for the Treatment of Alzheimer’s Disease
Source: Molecules. 2020 Feb 22;25(4):985. doi: 10.3390/molecules25040985 (PMC7070709; doi:10.3390/molecules25040985)
Supplement: Supplementary file 1 [file molecules-25-00985-s001.pdf]

Supplementary Information

Design, Synthesis and in vitro Evaluation of Hydroxybenzimidazole-Donepezil Analogues as Multitarget-Directed Ligands for the Treatment of Alzheimer's Disease

Sílvia Chaves [1], Simonetta Resta [1,2], Federica Rinaldo [1,2], Marina Costa [1], Romane Josselin [1], Karolina Gwizdala [1], Luca Piemontese [2], Vito Capriati [2,3], A. Raquel Pereira-Santos [4,5], Sandra M. Cardoso [4,5] and M. Amélia Santos [1,*]

[1] Centro de Química Estrutural and Departamento de Engenharia Química, Instituto Superior Técnico, Universidade de Lisboa, Av. Rovisco Pais, 1049-001 Lisboa, Portugal; silvia.chaves@tecnico.ulisboa.pt (S.C.); s.resta7@studenti.uniba.it (S.R.); f.rinaldo@studenti.uniba.it (F.R.); marinamcosta91@gmail.com (M.C.); josselinromane@gmail.com (R.J.); karo.gwizdala@gmail.com (K.G.);

[2] Dipartimento di Farmacia–Scienze del Farmaco, Università degli Studi di Bari "Aldo Moro", Via E. Orabona 4, I-70125 Bari, Italy; luca.piemontese@uniba.it (L.P.); vito.capriati@uniba.it (V.C.)

[3] Consorzio C.I.N.M.P.I.S., Via E. Orabona 4, I-70125 Bari, Italy

[4] CNC—Center for Neuroscience and Cell Biology, University of Coimbra, 3004-504 Coimbra, Portugal; araqpsantos@gmail.com (A.R.P.S.); cardoso.sandra.m@gmail.com (S.M.C.)

[5] Institute of Molecular and Cell Biology, Faculty of Medicine, University of Coimbra, 3000-548 Coimbra, Portugal,

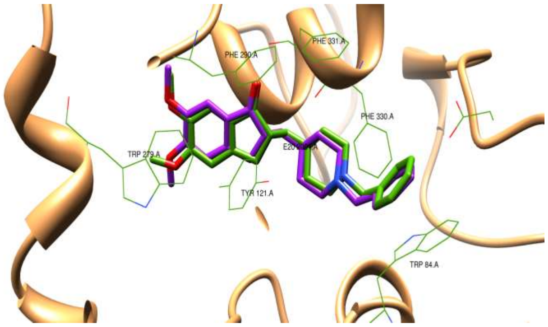

Figure S1. Redocking of original ligand (DNP) from PDB code 1EVE [1], inside the *TcAChE* active site, under the same simulation conditions used for the docking of the ligands in the present study.

[1] G. Kryger, I. Silman, J.L, Sussman, Structure Fold. Des. 7 (1999) 297-307
http://www.rcsb.org/pdb/explore/explore.do?structureId=1EVE
